# Mesenteric Lymph Node Recurrence of a Primary Colorectal Leiomyosarcoma

**DOI:** 10.1155/2020/6935834

**Published:** 2020-03-23

**Authors:** Amélie Beauchamp, Roy Hajjar, Sharmila Khullar, Mathieu Latour, Frank Schwenter, Herawaty Sebajang

**Affiliations:** ^1^Department of Surgery, Université de Montréal, Montreal, Canada; ^2^Digestive Surgery Service, Department of Surgery, Centre Hospitalier de l'Université de Montréal (CHUM), Montreal, Canada; ^3^Department of Pathology and Cellular Biology, Centre Hospitalier de l'Université de Montréal (CHUM), Montreal, Canada

## Abstract

Primary colorectal leiomyosarcoma is an excessively rare entity. It is associated with an aggressive behavior and typically favor hematogenous spread. The current standard of care is surgical resection. A 49-year-old patient presented with a 2-month history of fever. A PET-scan revealed a hypermetabolic mass in the transverse colon, and colonoscopy confirmed a tumor. A right hemicolectomy was performed. Histopathological diagnosis was of a leiomyosarcoma. Fourteen months after the surgery, a follow-up abdominal scan revealed a 2 cm mesenteric lymph node that was hypermetabolic on PET-scan. The mesenteric lymph node was resected and histopathology confirmed a leiomyosarcoma metastasis. This case opens the controversy on the management of rare lymph node recurrences in colorectal leiomyosarcoma.

## 1. Introduction

Colorectal adenocarcinoma is the most common histopathological type of colorectal cancer [1]. Primary leiomyosarcoma accounts for approximately 0.1% of all colorectal cancers [2–4]. This rare tumor subtype originates from smooth muscle cells and must be distinguished from gastrointestinal stromal tumors (GISTs) by immunohistochemical profiling [5]. It is associated with an aggressive behavior and a poor prognosis [1, 6, 7]. It is also associated with a high recurrence rate [4]. Leiomyosarcomas usually metastasize to the liver and lungs by hematogenous spread [8]. Lymphatic spread and nodal involvement are extremely rare [5]. Colorectal leiomyosarcoma is often initially misdiagnosed as adenocarcinoma and poses a major challenge to diagnosis, staging, and management [7]. It is most commonly diagnosed during the 5^th^ or 6^th^ decades of life and usually presents with nonspecific symptoms, such as abdominal pain and hematochezia [9]. The standard treatment of colorectal leiomyosarcoma consists of surgical resection [6, 10, 11]. The potential role of systemic treatment remains highly controversial, namely, when a nodal isolated recurrence is present [4]. This article describes an extremely rare case of mesenteric recurrence of a primary colorectal leiomyosarcoma and opens the controversy on the management of these recurrences.

## 2. Case Presentation

**Figure 1:**
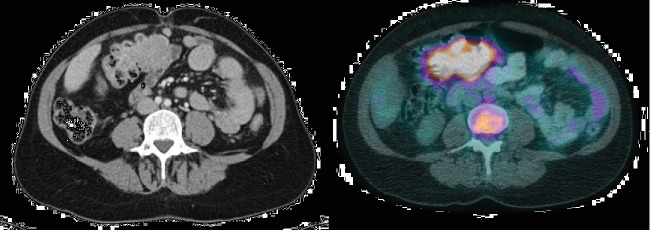
Transverse colon mass seen on abdominoplevic scan and PET-scan (FDG).

**Figure 2:**
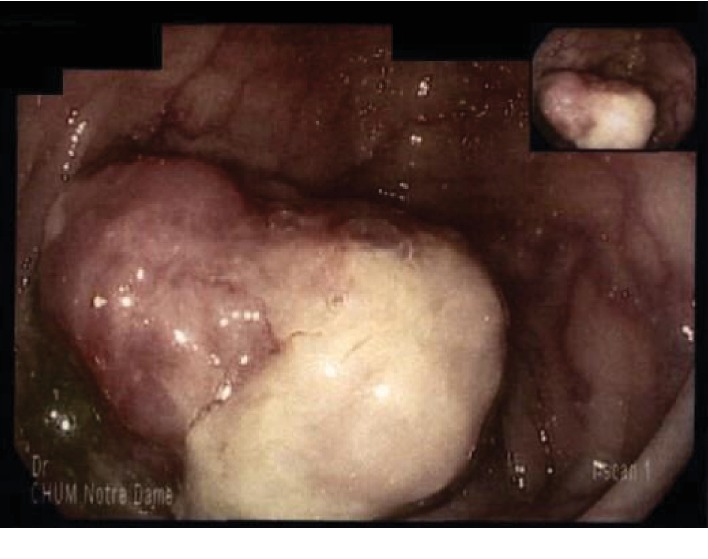
Transverse colon mass during colonoscopy.

A 49-year-old woman was evaluated for a fever of unknown origin that started 2 months prior to her visit. A wide panel of investigations and imaging testing was performed. An elevated level of C reactive protein (CRP) (160 mg/L) was noted. A positron-emission tomography (PET) scan revealed a hypermetabolic mass adjacent to the wall of the transverse colon (Figure 1). A colonoscopy was then performed, and a 3 cm indurated lesion was noted in the transverse colon (Figure 2). Biopsies were performed. Histopathological analysis revealed no neoplastic cells. A thoracic and abdominopelvic scan was performed and showed no signs of metastasis. Tumor markers, including CEA, CA 19-9, and CA125, were normal.

The patient underwent surgical exploration, and a right hemicolectomy was performed. Histopathological evaluation showed a 5 × 4 cm tumor composed of malignant fusiform cells. Immunohistochemical analysis was positive for vimentin, h-caldesmon, CD10, smooth muscle actin, and keratin AE1/AE3 and negative for desmin, WT1, EMA, PAX8, DOG1, CD117, CD34, keratin 5/6, S100, and p63. Ki-67 was estimated at 5-10%, and mitotic count was 10 per 10 high power fields (HPFs). Resection margins were negative. Fifteen lymph nodes were harvested and were free of metastasis. The patient recovered uneventfully from the surgery.

**Figure 3:**
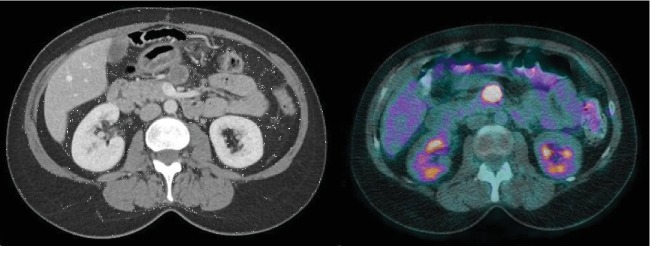
Follow-up abdominoplevic CT scan revealing a suspicious enlarged 2 cm mesenteric lymph node and PET-scan showing a high FDG uptake of the enlarged mesenteric lymph node.

The case was presented at the institutional cancer committee, and the joint decision was to not initiate adjuvant treatment. She had a normal follow-up abdominopelvic scan four months after the surgery. Fourteen months after the surgery, a second follow-up abdominopelvic CT scan showed a suspicious enlarged 2 cm mesenteric lymph node. It was described as necrotic and located under the pancreas. This lymph node showed hypermetabolism on the PET-scan with a maximum standardized FDG-uptake value (SUV) of 7.1 (Figure 3).

**Figure 4:**
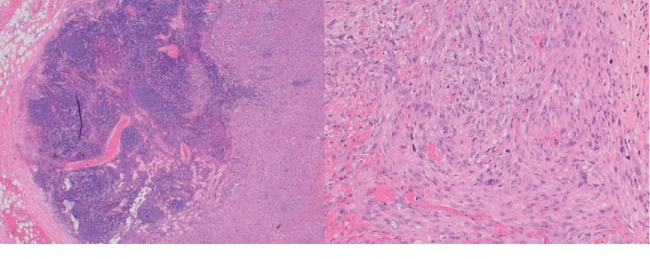
Hematoxylin and eosin stain, enlarged 20x, lymph node infiltrated with tumor cells and hematoxylin and eosin stain, enlarged 100x, findings suggestive of leiomyosarcoma found within the mesenteric lymph node.

**Figure 5:**
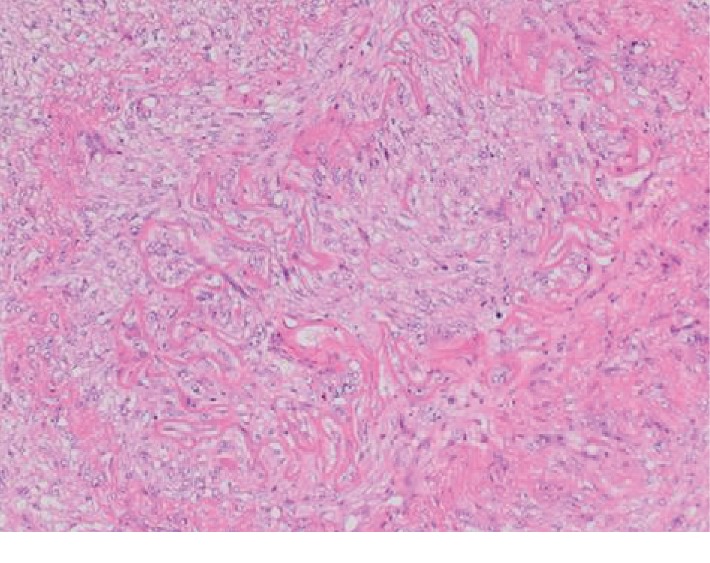
Hematoxylin and eosin stain, enlarged 100x, vascular involvement of the tumor within the mesenteric lymph node.

A laparotomy was performed to allow adequate access to the lymph node. A 3 cm mass was identified between the superior mesenteric artery and the splenic vein and was resected. Histopathology showed a neoplastic proliferation of pleomorphic spindle cells arranged in fascicular and storiform patterns, compatible with a leiomyosarcoma (Figure 4). One lymph node out of seven (1/7) was found to harbor a metastatic focus, which seemed to stem from adjacent vascular invasion (Figure 5). Resection margins were negative. The postoperative period was complicated by chylous ascites, which was detected due to the presence of a Hemovac drain installed during the surgery. This complication was most probably caused by extensive dissection that was required to access the enlarged lymph. The drain was removed 15 days later during a follow-up appointment.

This rare case was presented again at the institutional cancer committee, and the decision was to proceed with clinical follow-up without adjuvant chemotherapy. Eight months after the surgery, the patient is disease free.

## 3. Discussion

Colorectal leiomyosarcoma is an exceedingly rare entity, accounting for approximately 0.1% of all colorectal cancers [2–4]. Other types of sarcomas that can potentially affect the colon and rectum include fibrosarcomas and angiosarcomas. Leiomyosarcomas account for more than 95% of colorectal sarcomas [1]. Gastrointestinal leiomyosarcomas affect most commonly the stomach, followed by the small bowel, rectum, and colon. Rectal leiomyosarcomas are twice more common than colonic leiomyosarcomas [8]. This entity can also affect the uterus and the retroperitoneum [7]. A few cases of appendiceal leiomyosarcomas have been reported as well [12]. Clinically, leiomyosarcomas represent a major diagnostic challenge. In addition to their scarcity compared to colorectal adenocarcinoma, initial biopsies are often negative or inconclusive [13–15]. Leiomyosarcomas are thus often mistakenly identified as adenocarcinomas until surgical resection and final pathological analysis. In the case presented here, the primary tumor and subsequent metastasis were initially diagnosed and characterized through high FDG uptake on the PET-scan. Although this imaging method may be an adequate adjunct to other tests, it remains nonspecific in the identification of a leiomyosarcoma. Histological analysis remains essential.

The diagnosis of leiomyosarcoma is more commonly made after the evaluation of the surgical specimen. Leiomyosarcomas are malignant tumors derived from smooth muscle cells of the colonic muscularis propria [5]. Tumor cells are usually spindled, with brightly eosinophilic cytoplasm and cigar-shaped, blunt-ended nuclei. They are arranged in perpendicularly oriented fascicles. Pleomorphism and tumor necrosis are often present [11]. Immunohistochemically, these cells are usually positive for smooth muscle actin, desmin, and h-caldesmon. CD117 may be expressed but is not accompanied by KIT or PDGFRA gene mutations. Protein S100, CD34, and DOG1 are negative [5, 11, 15]. Leiomyosarcomas traditionally metastasize via hematogenous spread, with lymphatic spread being anecdotic [2, 12].

The main histological differential diagnosis of a primary colonic leiomyosarcoma is a gastrointestinal stromal tumor (GIST) [11]. GISTs, which originate from interstitial cells of Cajal, are composed of uniform spindled or epithelioid cells arranged in lobules [16, 17]. These cells have eosinophilic cytoplasm and often display cytoplasmic vacuoles. The so-called “skeinoid” fibers, representing coarse, wire-like, haphazardly arranged collagen bundles, are sometimes observed [17]. Nuclear pleomorphism and tumor necrosis, when present, portend a poor prognosis [17]. In most instances, GIST cells express a combination of CD34, CD117, DOG1, and h-caldesmon [17, 18]. They may express protein S100 and smooth muscle actin, but not desmin [16, 17]. In approximately half of the patients, these tumors follow an indolent course, but when malignant, they should be readily treated with tyrosine kinase inhibitors such as imatinib mesylate [19].

Complete resection of colonic leiomyosarcomas is rarely described in the literature, with less than 20 cases reported [5]. The immunohistochemical profile of the case presented here is highly compatible with a true leiomyosarcoma. The initial negative biopsy in this case also highlights the challenge of obtaining a pathological diagnosis for intramural tumors prior to surgery.

The most widely accepted and performed treatment with colonic leiomyosarcomas is surgical excision [6, 10, 11]. The goal of surgery is to achieve complete microscopic removal of tumor with negative margins [11]. To our knowledge, due to the rarity of this presentation, there are no guidelines detailing the optimal extent of lymphadenectomy [11]. In colon adenocarcinomas, a standard lymphadenectomy is recommended, which include the harvest of at least 12 lymph nodes following the vascularization of the affected colonic segment, combined with the removal of macroscopically suspicious lymph nodes [20]. It is unclear if these guidelines could also be applied to soft tissue sarcomas of the colon, because lymph node involvement in these cases is extremely rare. For the same reason, extended lymphadenectomy is not routinely recommended [10, 12]. In the case described here, in which standard guidelines for lymphadenectomy in classic adenocarcinoma were followed, none of the 15 lymph nodes removed during the initial surgery were positive. Nonetheless, it seems logical to follow these guidelines to facilitate “en bloc” resection and because the mesenteric lymph nodes are removed along with the vessels supplying the tumor [11]. Additionally, because of the high local recurrence rate, a more extensive local resection seems safer to ensure negative margins of resection [3]. In this case, there was no indication that a more extensive lymphadenectomy was initially indicated. As for the mesenteric recurrence, a more targeted resection of the enlarged lymph node was conducted and 7 lymph nodes were removed. There are no guidelines addressing the management of mesenteric recurrence of leiomyosarcomas. Thus, it is unclear if a more extensive resection would have been beneficial.

The role of systemic therapy for colorectal leiomyosarcoma is controversial. Most authors do not recommend initiation of a systemic chemotherapeutic agent, as the majority of leiomyosarcomas are chemoresistant [3, 21]. Studies addressing the possible benefits of systemic therapy provide furthermore inconsistent results [4, 10]. Some systemic agents have been proposed or studied, like doxorubicin, ifosfamide, dacarbazine, gemcitabine, and docetaxel [5, 22]. The utility of these agents was assessed in soft tissues or bone sarcomas, but not in colorectal sarcomas specifically [23, 24]. The National Cancer Database (NCDB) shows that approximately 15% of patients with colorectal sarcomas undergo chemotherapy. These patients are more likely to be younger, to have positive lymph nodes, and to not undergo surgery [10]. In the case presented here, a multidisciplinary tumor board decided against the use of a systemic agent, even after the mesenteric lymph node recurrence. The complexity and rarity of these particular cases warrant a discussion in multidisciplinary meetings involving surgeons, pathologists, oncologists, and radiation oncologists.

Radiotherapy is not recommended for the treatment of colorectal sarcomas, mainly because these tumors are frequently radioresistant [8, 13]. The NCDB review showed that approximately 12% of patients with colorectal sarcomas received radiotherapy. They were more likely to be younger, to have positive resection margins after the surgery, and to have a primary tumor located in the rectum. These patients showed no differences in terms of survival compared with surgery only [10]. It has been suggested that radiotherapy for rectal leiomyosarcomas could lower the risk of local recurrence [3]. However, this premise remains controversial and not widely accepted.

Colorectal leiomyosarcomas are associated with limited survival and a poor prognosis. The overall 5-year survival is between 20 and 44% [4, 5, 25]. Factors associated with poorer prognosis include a tumor size greater than 5 cm and a high mitotic count (>10/50HPFs) [2, 6, 26–28]. Mitotic count seems to be a good indicator of metastatic potential [8]. To our knowledge, there is no study evaluating the impact of lymph node involvement on prognosis and survival. Moreover, colorectal leiomyosarcomas are associated with a high recurrence rate. According to some studies, half of the recurrences are local and the other half are distant [3]. Distant metastases are most commonly found in the liver, the peritoneum, and the lungs [2, 29]. Lymph node recurrence is very rarely described, because sarcomas intrinsically favor hematogenous over lymphatic spread [5].

## 4. Conclusion

We presented here a rare case of mesenteric lymph node recurrence of primary colorectal leiomyosarcoma. These are rare entities associated with an aggressive behavior and represent a diagnostic challenge. Current standard of care is surgery alone. More studies are required to evaluate the impact on prognosis and subsequent management of nodal involvement in colorectal sarcomas.
